# Social Isolation and Loneliness: Develop a More Robust Evidence Base

**DOI:** 10.1093/geroni/igaa057.2513

**Published:** 2020-12-16

**Authors:** Holt-Lunstad Julianne

**Affiliations:** Brigham Young University, Provo, Utah, United States

## Abstract

This paper reviews the evidence base for the health impacts of SIL on older adults, the risk factors, and the potential moderators and mediators of those relationships. Substantial evidence indicates that SIL are associated with physical, cognitive, and psychological morbidity; health-related behaviors; and health-related quality of life. Social isolation in particular is associated with a significantly increased risk of premature mortality from all causes. The evidence base for interventions in the clinical setting is also reviewed. Finally, the report’s specific recommendations are discussed, including the need for basic science research, increased funding, key elements in the design and evaluation of interventions, and gap areas (including trends among current younger adults and approaches for specific understudied groups of at-risk older adults). Part of a symposium sponsored by Loneliness and Social Isolation Interest Group.

